# Malaria Prevalence and Distribution of *Plasmodium* Species in Southern Region of Ethiopia

**DOI:** 10.1155/2022/5665660

**Published:** 2022-06-23

**Authors:** Anmut Assemie

**Affiliations:** Department of Biology (Entomology), Wachemo University, Pox, 667 Hossana, Ethiopia

## Abstract

Malaria is caused by Plasmodium species and transmitted by Anopheles mosquitoes, which is the most common medical concern all over the world, including in Ethiopia. The current systematic review's goal was to determine the overall malaria prevalence and Plasmodium species distribution in Ethiopia's southern area. To achieve these objectives, 716 articles were manually searched from online databases such as PubMed, Scopus, Google Scholar, Science Direct, and Web of Science. The pooled metalogistic regression was calculated with the STATA 16 software to present the pooled prevalence with a 95% confidence interval (CI). Eighteen full-text articles met the inclusion criteria and were included in the study, out of the 716 studies initially identified. The majority of the articles in the systematic review used a cross-sectional study design, with sample sizes ranging from 160 to 583,668 participants. The study's lowest and highest malaria prevalence was 0.93% and 82.84%, respectively. During the current systematic review, the estimated malaria prevalence was 19.19% (95% CI: 14.67–23.70). There were 263,476 positive individuals in the study, accounting for 148,734, 106,946, and 7,796 cases of P. falciparum, P. vivax, and mixed infections, respectively. The overall prevalence of P. falciparum and P. vivax was 8.97% (95% CI: 6.31, 11.63) and 7.94% (95% CI: 6.56, 9.33), respectively. According to the systematic review, the most predominant Plasmodium species responsible for malaria disease in the study area was P. falciparum. The highest malaria rates were found in this systematic review. In the systematic review, P. falciparum was the most dominant Plasmodium species that was responsible for malaria disease in the study area. This systematic review indicates the highest malaria prevalence in the southern regions of Ethiopia. Therefore, existing malaria prevention and control strategies in the southern region of Ethiopia should be revised.

## 1. Introduction

Malaria is a mosquito-borne disease caused by five *Plasmodium* parasites [1] and transmitted by infective female *Anopheles* mosquito bites [1–3]. *Plasmodium falciparum* and *Plasmodium vivax* are the most widely distributed and well-known malaria-causing species in Ethiopia, accounting for 60% and 40 percent of cases, respectively [4].

Malaria disease transmission is influenced by environmental factors. The key factors that affect the spread of malaria infections include topography, rainfall, climate, and socioeconomic conditions of the population [5–8]. In Ethiopia, the transmission of malaria is unstable and seasonal. September to December and April to May were the two malaria transmission season in Ethiopia [9].

From 2001 to 2011, the average rate of new malaria cases confirmed in Ethiopia declined from 78,325 in 2001-2005 to 30,780 in 2011. The slide positivity rate was 24% in 2001 and 11% in 2011 [10]. Malaria prevalence declined from 2.8 million in 1990 to 621,345 in 2015, according to a 2015 study. Malaria-related deaths reduce from 30,323 in 1990 to 1561 in 2015. Over the last 25 years, there has been a 94.8% decrease [11]. In Ethiopia, however, new malaria cases were 2,927,266 in 2016, with 4,782 deaths [12].

Ethiopia is currently extending the 2020 malaria eradication strategy to 2030. As a result, the goal of this review was to assess the prevalence of malaria and the distribution of *Plasmodium* species in the Southern region of Ethiopia. In the Southern region of Ethiopia, different studies were conducted to know the prevalence rate of malaria with great differences and varying findings. But, there is no previous systematic review that indicates the prevalence of malaria and the distribution of *Plasmodium* species in the regions. Due to this, the current review was designed to know the prevalence of malaria and the distribution of *Plasmodium* species in the region. The finding of the review has significant value in malaria prevention and control programs at the country and international levels. It is also useful to evaluate the progress of the region towards achieving the regional and national targets and to take immediate action in the planning and implementation of prevention and control strategies.

## 2. Methods

### 2.1. Description of the Study Area

The Southern region of Ethiopia is located in the southern and southwestern parts of the country. It lies between 4.43-8.53 degrees' North latitude and 34.88-39.14 degrees' East longitude and is bordered by Kenya in the South, the Sudan in the South West, Gambella region in the North West and surrounded by the Oromia region in the North West, North, and East. Based on the 2007 census conducted by the Central Statistical Agency of Ethiopia, the Southern region has an estimated total population of 14,929,548, of whom 7,425,918 were men and 7,503,630 women. The total area of the region is about 110,932 km^2^. The region features a variety of climatic and agroecological zones, including Kefil Bereha, Kolla, Woina Dega, Dega, and Wurch, which are located between 350 and 4200 meters above sea level. Kolla and Woina Dega make up the majority of the region, with Kolla and Woina Dega accounting for around 80% of the total (JICA. 2012). The majority of the land is malarious because it is less than 2,000 meters above sea level. Currently, Ethiopia's population is estimated to be over 90 million, with approximately 68% residing in malaria-prone areas, including the Southern region [13]. All the articles which are included in the systematic review were done in the Southern region of Ethiopia.

### 2.2. Literature Search Strategy

The search articles comprised different study designs (cross-sectional surveys and longitudinal studies) with reported malaria prevalence results (positive-negative cases and parasites) in well-defined populations. Articles were searched without time restriction by using the key terms “malaria prevalence,” “burden of malaria,” “distribution of *Plasmodium* species,” “malaria parasites,” and “malaria epidemiology in Ethiopia.”

From the total reviewed articles, those studies published in peer-reviewed journals such as PubMed, Scopus, Google Scholar, Science Direct, Web of Science, and other related journals that report the prevalence of malaria in the Southern region of Ethiopia, were included in the review.

All studies included in the systematic review were original research articles published in English and contained the basic information concerning sample size, diagnostic methods, prevalence, and status of malaria infection among all target populations, pregnant women, and children in different parts of the Southern region of Ethiopia.

The study conducted among unknown malaria detection methods, an unknown sample size, and a lack of clear figures about infected cases were excluded in this review. After searching PubMed, Google Scholar, Science Direct, Web of Science, and Scopus using keywords, articles were screened by title and abstract to transfer the articles in the full-text review. The quality of articles was assessed using Joana Brigg's Institute (JBI) critical appraisal checklist for simple prevalence [14].

The data extraction protocol was developed by the researcher and evaluated by Ass. Professor Wubetu Barude and Ass. Professor Ritbano Ahmed. This extraction protocol consists of the name of the first author, year of publication, study area, study group, study design, target sample, sample size, the prevalence of malaria, prevalence of *P. falciparum*, the prevalence of *P. vivax,* the prevalence of mixed infection, and type of diagnostic method used. Once the data were extracted using Microsoft Excel, data manipulation and statistical analysis were done using STATA 16 version software.

## 3. Results

### 3.1. Description of Study Articles

**Figure 1:**
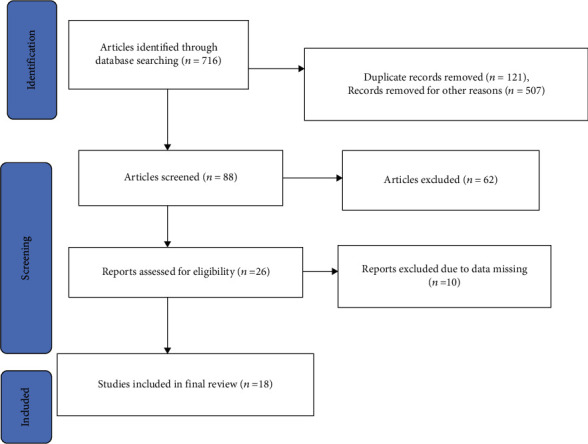
Flowchart diagram that shows the selection of studies included.

The systematic review contains published articles on the prevalence of malaria among patients in the Southern region. A total of 716 articles were retrieved from online databases using manual searching. Out of these studies, 121 articles were excluded due to duplication records. From the remaining 595 articles, 507 articles were excluded due to their study area, titles, and abstracts. Due to this, only 88 articles were eligible for full-text assessment which is done in different parts of Ethiopia. Of the 88 eligible articles, 62 studies were excluded from the review because they are done out in the Southern region of Ethiopia. Only twenty-six full-text articles were screened which were done in the Southern region of Ethiopia. Finally, eighteen full-text articles were included in this systematic review because the remaining ten are not eligible and excluded in the final systematic review due to a lack of clear data, such as the prevalence of malaria, positive cases, total population, and study design as shown in Figure 1.

### 3.2. Characteristics of the Reviewed Articles

A total of eighteen articles were included in this systematic review. The papers which were considered for this review, including the author's name, study design, target population, sample size, prevalence of malaria, distribution of *Plasmodium* species, statistical methods used, software used for computation and carried out in different parts of Southern region of Ethiopia as indicated in Table 1 were used.

From the total articles reviewed, twelve studies used a cross-sectional design, five used retrospectives, and one study used a longitudinal research design. Out of all included studies, a study conducted in Arbaminch General Hospital and Arbaminch health center Southern Ethiopia had the least sample size with 160 study participants [15]. However, another study conducted in Halaba special district in Southern Ethiopia had the highest sample size with 583,668 study participants [16]. Thirteen studies were used for microscopic examination, four used mixed, and one used rapid diagnostic tests examination. The systematic review indicated that microscopic tests become the more come to determine the malaria case. The included article in the systematic review was done in the time interval between 2003 and 2019 as indicated in Table 1. The target population of the systematic review was Children less than 15 years old [17–20], pregnant [11, 21], and the remaining Suspected. As indicated in Table 1, the prevalence of malaria in the primary studies ranges from 0.93% [22] to 82.84% which indicates the severity of malaria in the Southern region [10]. The majority of the primary studies indicate a high prevalence of malaria and distribution of *Plasmodium* species [10, 15, 18, 23–26].

### 3.3. Prevalence of Malaria Disease

**Figure 2:**
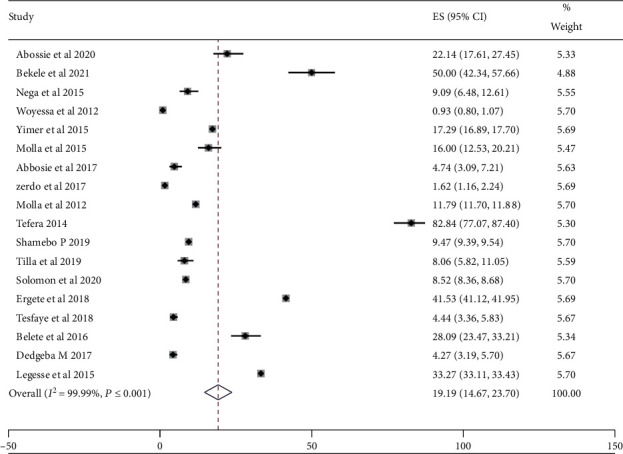
The pooled prevalence of malaria from the random-effects model.

In the reviewed articles, a total of 1,622,356 study participants were tested for malaria infection from the eligible articles. Among these, 263,476 were positive for malaria infection. The overall malaria prevalence in the aspect of gender preference, men were more affected than females, which accounts for 136,683 (51.87%) males and 126,793 (48.12%) females. Concerning age groups, those individuals whose ages were greater than 15 were highly affected by malaria [8, 11, 12, 21, 24, 25]. But, other four studies indicated that children in the age group of ≤15 years were the most affected group by malaria [17–19, 27]. The prevalence of malaria among the primary studies ranges from 0.93% in Butajira [22] to 82.84% in Halaba [10] as shown in Table 2. The overall estimated prevalence of malaria was 19.19% (95% CI:14.67, 23.70) as indicated in Figure 2. High heterogeneity (*I*^2^ = 99.99% and *p* value ≤ 0.001) across studies was observed in the analysis.

### 3.4. The Abundance of *Plasmodium* Species

**Figure 3:**
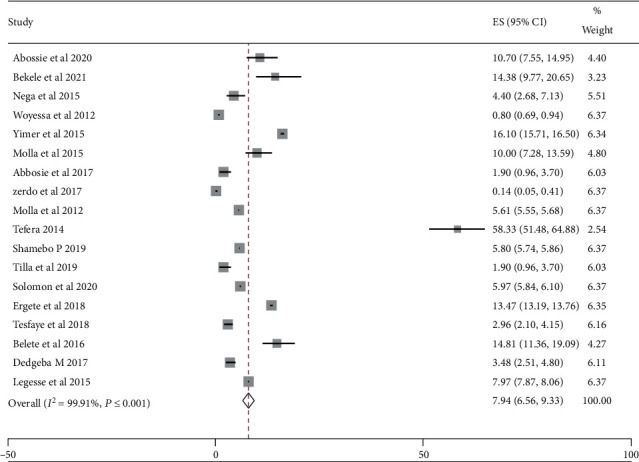
The pooled prevalence of Plasmodium vivax species from the random-effects model.

**Figure 4:**
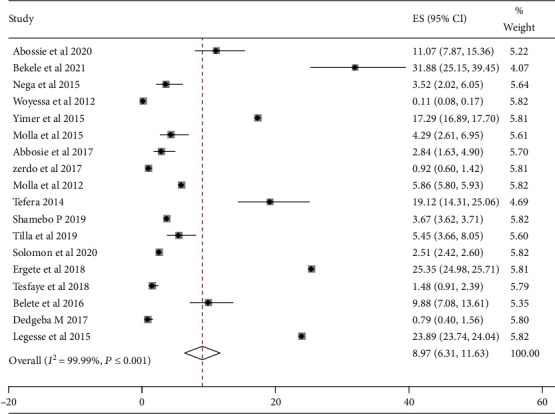
The pooled prevalence of P. falciparum species from the random-effects model.

In the current systematic review*, Plasmodium falciparum* and *Plasmodium vivax* were the two most predominant and widely occurred parasites of *Plasmodium* species responsible for malaria cases in the Southern region of Ethiopia. *Plasmodium falciparum* and *P. vivax* coexist as major parasite species in the Southern region of Ethiopia. *Plasmodium falciparum* was the dominant parasite that accounted for 8.97% of the pooled prevalence estimate with 95% (CI: 6.31, 11.63) and *I*^2^ = 99.9% between-study heterogeneity. *P. vivax* is the second most dominant malaria parasite in the Southern region of Ethiopia which accounted for 7.94% (95% CI: 6.56, 9.33) as shown in Figures 3 and 4, respectively.

### 3.5. Distribution of *Plasmodium* Species

As indicated in Table 2, from the total examined, 263,476 were found positive for malaria parasites. All studies included in this review reported the prevalence of *P. vivax* and *P. falciparum* infection, while the prevalence of mixed infections was not reported in four studies. Out of these positive (40.59%), 106,946 were positive for *P. vivax*. *Plasmodium falciparum* accounts for (56.45%) of 148,734 total positive cases and the remaining (2.96%) 7,796 of them had mixed infection. In these systematic reviews, the highest and lowest distributions of *Plasmodium falciparum* was 71.79% and 12.36%, respectively. In all reviewed journals, there was a distribution of mixed *plasmodium* parasites except those studied by [8, 16, 17, 24]. Regarding gender, the distribution of *Plasmodium* species was more common in males than females. *Plasmodium vivax* was more dominate in the age greater than 15 years [8, 10, 12, 19, 21, 23], but *Plasmodium falciparum* was dominant in the child whose age less than 15 years [17, 18, 20].

## 4. Discussion

The present systematic review assessed the pooled prevalence of malaria and distribution of *Plasmodium* species in the Southern region of Ethiopia. This systematic review was conducted using eighteen full-text articles to determine the pooled prevalence of malaria among individuals in the Southern region of Ethiopia.

Malaria causes serious complications in humans, such as severe anemia, acute renal failure, hypoglycemia [29], loss of productivity, and school absenteeism [9]. Due to these, accurate malaria prevalence information is vital for proper diagnosis, treatment, prevention, and policy preparation [13].

In the current systematic review, the pooled prevalence of malaria in the Southern region of Ethiopia was 19.19%. This is greater than the systematic review and meta-analysis conducted on malaria prevalence among adults, children, and pregnant women in Ethiopia, which results in 13.61%, 9.07%, and 12.72%, respectively [30–32].

This review is nearly similar to the research conducted in Dilla town and the surrounding rural areas, Gedeo Zone, Southern Ethiopia, which was 16% [27]. On the other hand, the result of this study was much lower than the previous research studies reported in Ethiopia among all age groups [10, 15, 23–26]. The most likely reason for this variation is that some of the studies were obtained from the high malaria endemic areas of the country, while others were obtained from medium and low malaria-risk areas.

The overall prevalence of *P. falciparum* (9.17%), *P. vivax* (6.60%), and mixed (0.48%) parasites were found in proportions of 56.45%, 40.59%, and 2.96%, respectively. The results of this systematic review showed that *P. falciparum* and *P. vivax* have around 15% variation but greater variation compared to the mixed parasites. The overall estimated distribution of *P. falciparum* and *P. vivax* in the current systematic review was similar to nine of the articles included in the review [11, 15, 17, 18, 20, 24–28] and contradict with other nine of the reviewed articles [8, 10, 12, 16, 19, 21–23, 27]. The variation in the prevalence of malaria might be related to the difference in the type of laboratory method used, methods of data collection, time of the study, and the difficulty of implementing the existing malaria prevention and control measures practice. The distribution of *Plasmodium* species in this systematic review is also similar to studies conducted in Bichena Primary Hospital, Amhara Region [33], Tselemti Wereda, North Ethiopia [34], Gambella University [35], and Kalala Health Center in Haro Limmu Woreda, East Wollega Zone [36]. Similarly, the overall prevalence of *P. falciparum* was 8.97% (95% CI: 6.31, 11.63), *P*. *vivax* 7.94% (95% CI: 6.56, 9.33) parasites resulted in a proportion of 56.45% and 40.59%, respectively. These results were nearly similar to the previous predictions of *P. falciparum* (60%) and *P*. *vivax* (40%) in Ethiopia [4, 10, 37, 38].

The estimated malaria prevalence over time is not consistent. This is because malaria infection in Ethiopia is highly variable and unstable, and the occurrence of epidemics over several locations (agroecological regions) of the country. Malaria prevalence in Ethiopia is seasonal [39–42]. The two seasons of malaria transmission in Ethiopia coincides with the peak agricultural activities [37, 39, 41, 43]. Due to this reason, the country's economy is significantly affected. Not only this but also malaria transmission is also highly variable [39–42]. This could be due to the presence of different topographic platforms that control the multiplication rate and diversity of the *Anopheles* mosquito vectors.

The studies included in this systematic review showed high heterogeneity in malaria prevalence. The possible source of heterogeneity might be related to the seasons in which each of the included studies was conducted because some of the studies were conducted during the high malaria transmission seasons, whereas the rest of included studies were done during the low malaria transmission seasons and also the methods of diagnosis.

## 5. Conclusions

The overall estimated prevalence of malaria was 19.19% (95% CI: 14.67, 23.70). The majority of the studies in this review met the quality criteria, and there was no publication bias. Based on the included articles in the review, microscopic diagnosis of malaria cases was common in the study area. The pooled distribution of *Plasmodium* species agrees with the national figures. This systematic review showed a high prevalence of malaria in the Southern regions of Ethiopia. Therefore, previous prevention and control strategies could be revised and new strategies implemented. In addition, to meet the WHO's 2030 goal, the region requires independent and stand-alone malaria prevention and control task forces.

## Figures and Tables

**Table 1:** Characteristics of those included reviewed articles.

Authors	Study period	Sample size	Target population	Study design	Diagnostic methods	Positive	Prevalence	Software
[18]	April-May 2017	271	Children <5 year	Cross-sectional	Microscopy	60	22.22	SPSS
[15]	Oct-Dec 2016	160	Suspected	Cross-sectional	Mixed	80	50	SPSS
[21]	Apr-June 2013	341	Pregnant women	Cross-sectional	Mixed	31	9.09	SPSS
[22]	Oct/2008-June/2010	19,207	Suspected	Cross-sectional	Microscopy	178	0.93	SPSS, STATA
[26]	Feb/2008-Dec/2012	34,060	Suspected	Cross-sectional	Microscopy	11,523	33.83	SPSS
[27]	October-Dec/2014	350	Suspected	Cross-sectional	Microscopy	56	16	SPSS
[17]	Dec/2014-Feb/2015	422	Children 6 to 15 years	Cross-sectional	Mixed	20	4.74	SPSS
[20]	October-Dec 2019	2,167	School-aged children	Cross-sectional	RDT	35	1.62	R
[28]	Jan/2012-Dec/2019	485,414	Suspected	Retrospective	Microscopy	57,228	11.79	STATA
[10]	April-June 2009	204	Suspected	Cross-sectional	Microscopy	169	82.84	SPSS
[16]	2003-2017	583,668	Suspected	Retrospective	Microscopy	55,252	9.46	SPSS
[11]	April-May 2016	422	Pregnant women	Cross-sectional	Microscopy	34	8.05	SPSS
[12]	2015-2018	121,230	Suspected	Retrospective	Microscopy	10,379	8.56	SPSS
[24]	2008-2014	54,160	Suspected	*Retrospective*	Mixed	22,494	41.53	SPSS
[8]	Oct-Dec 2006	1,082	Suspected	Longitudinal	Microscopy	48	4.44	SPSS
[23]	May-June 2016	324	Suspected	Cross-sectional	Microscopy	91	28.08	SPSS
[19]	Sept 2015-Jan 2016	1,007	Febrile children	Cross-sectional	Microscopy	43	4.27	SPSS
[25]	Mar-Aug 2014	317,867	Suspected	Retrospective	Microscopy	105,755	33.27	SPSS
Total		1,622,356				263,476	19.19	

**Table 2:** Distributions of *Plasmodium* species in the Southern region of Ethiopia.

Reference	Total participant	Positive	Plasmodium species			Sex	
			P. falciparum	P. vivax	Mixed	Male	Female
[18]	271	60	30	29	1	32	28
[15]	160	80	51	23	6	50	30
[21]	341	31	12	15	4	0	31
[22]	19,207	178	22	154	2	99	79
[26]	34,060	11,523	5,889	5,484	150	6,023	5,500
[27]	350	56	15	35	6	33	23
[17]	422	20	12	8	0	10	10
[20]	2,167	35	20	3	12	19	16
[28]	485,414	57,228	28,468	27,235	1,525	29,480	27,748
[10]	204	169	39	119	11	72	97
[16]	583,668	55,252	21,397	33,855	0	27,540	27,712
[11]	422	34	23	8	3	0	34
[12]	121,230	10,379	3,044	7,237	98	5,336	5,043
[24]	54,160	22,494	13,727	7,297	1,470	14,229	8,265
[8]	1,082	48	16	32	0	25	23
[23]	324	91	32	48	11	51	40
[19]	1,007	43	8	35	0	22	21
[25]	317,867	105,755	75,929	25, 329	4497	53,662	52,093
Total	1,622,356	263,476	148,734	106,946	7,796	136,683	126,793

## Data Availability

The data that support this systematic review is available in the manuscript and in the primary studies which were used to support the development and completion of this systematic review.
